# Inverted U-Shaped Association of Soluble Transferrin Receptor Concentrations with Risks of Cardiovascular Diseases in Overweight Individuals: A Cross-Sectional Study

**DOI:** 10.31083/j.rcm2512439

**Published:** 2024-12-16

**Authors:** Xiao Hu, Jing Xu, Yang Gu

**Affiliations:** ^1^Department of Cardiology, The Affiliated Huaian No.1 People’s Hospital of Nanjing Medical University, 223300 Huaian, Jiangsu, China; ^2^Department of Respiratory and Critical Care Medicine, The Affiliated Huaian No.1 People’s Hospital of Nanjing Medical University, 223300 Huaian, Jiangsu, China

**Keywords:** soluble transferrin receptor, overweight, cardiovascular disease, National Health and Nutrition Examination Survey

## Abstract

**Background:**

Iron metabolism may play a role in cardiovascular disease (CVD) pathogenesis. The association between iron metabolism and CVD has yet to be fully investigated. This study evaluated whether iron metabolism was associated with CVD risk and whether the body mass index (BMI) of US adults varied the association.

**Methods:**

A cross-sectional study was performed using the National Health and Nutrition Examination Survey (NHANES), conducted from 2017 to 2018. Generalized additive models (GAMs) and multivariable logistic regression were adopted to analyze the association between iron metabolism (serum iron (SI), serum ferritin (SF), transferrin saturation (TSAT), and soluble transferrin receptor (sTfR)) and CVD risk. Further, stratified analysis was conducted to identify patients with high CVD risk.

**Results:**

Participants with CVD tended to have significantly increased levels of sTfR (*p* < 0.001) and decreased levels of TSAT (*p* < 0.001) and SI (*p* < 0.001). After adjusting for confounding factors, sTfR levels had a significant positive association with CVD risk (Q1 as reference, Q4 odds ratio (OR) 2.1, 95% CI 1.54–2.87, *p* < 0.001). Notably, the association between sTfR and CVD risk differed in the BMI subgroup (*p* for interaction < 0.05). We identified an inverted U-shaped relationship between sTfR and the CVD risk in the group of overweight individuals (non-linear *p* < 0.001). When the sTfR level was below the turning point (sTfR = 5.35 mg/L), a per unit increase in the sTfR level was correlated with a 78% greater adjusted OR of CVD risk (OR, 1.78 [1.44, 2.19]).

**Conclusions:**

Increased sTfR levels were non-linearly related to the CVD risk in the overweight population.

## 1. Introduction

Cardiovascular disease (CVD) is the major contributor to premature deaths among adults and increased risk of disability [1]. Over the past 50 years*, *current efforts have focused on risk assessment and mitigation. Despite substantial advances, risk prediction remains a central part of disease prevention due to the persistently high incidence of cardiovascular disease.

As a result of its high energy requirements, the heart is the most mitochondrial-rich organ in the body. While iron plays a crucial role in DNA synthesis, vital metabolic reactions, and cellular respiration, alterations in iron status continue to pose a significant health threat [2]. Iron deficiency (ID) can cause anemia, whereas excess iron can lead to cardiovascular disease [3]. Atherosclerosis development, myocardial infarction, and heart failure associated with iron overload are significant causes of morbidity and mortality in people with this condition [4]. Meanwhile, an iron overload could lead to ferroptosis, a novel form of programmed cell death. Several biomarkers can demonstrate iron status, including serum ferritin (SF), serum iron (SI), transferrin saturation (TSAT), and soluble transferrin receptor (sTfR).

Recent studies have suggested that iron metabolism dysregulation could play a role in CVD [5]. Although some clinical and epidemiological studies have demonstrated an association between iron metabolism biomarkers and CVD, the results remained inconsistent and/or inconclusive. Recently, Meng *et al*. [6] reported that decreased SI concentration levels could be a potential predictive biomarker of coronary atherosclerosis. Another research discovered that SF is positively associated with CVD [7]. A meta-analysis of 17 studies showed that increased TSAT was inversely related to coronary heart disease (CHD) [8]. A negative association also existed between SI and CHD/myocardial infarction after the removal of one study. However, no significant association was observed between the other iron status markers and CHD. Regarding cardiovascular morbidity, a study that enrolled 2874 participants showed no relationship between SF and the risk of CVD during a 10-year follow-up [9]. The relative importance of ID for coronary artery disease (CAD) mortality has also been demonstrated previously [10], indicating a J-shaped curve for the association between SF and mortality, suggesting that both low and high SF concentrations present a poor prognosis in type 2 diabetes patients with CAD.

There has long been recognition that obesity is a distinct risk factor for CVD. BMI (body mass index) indicates weight-related nutritional status and fat storage in the body [11]. Nonetheless, evidence suggesting an association between iron metabolism biomarkers and CVD based on BMI categories is, to our knowledge, scarce. The present study hypothesized that iron status markers associated with different categories of BMI may have distinct effects on the incidence of CVD. Therefore, this study aimed to systematically investigate the association between biomarkers of iron metabolism and CVD and, more significantly, to evaluate the potential modification of BMI in this relationship among US adults in the 2017–2018 National Health and Nutrition Examination Survey (NHANES).

## 2. Methods

### 2.1 Study Participants

**Fig. 1.**
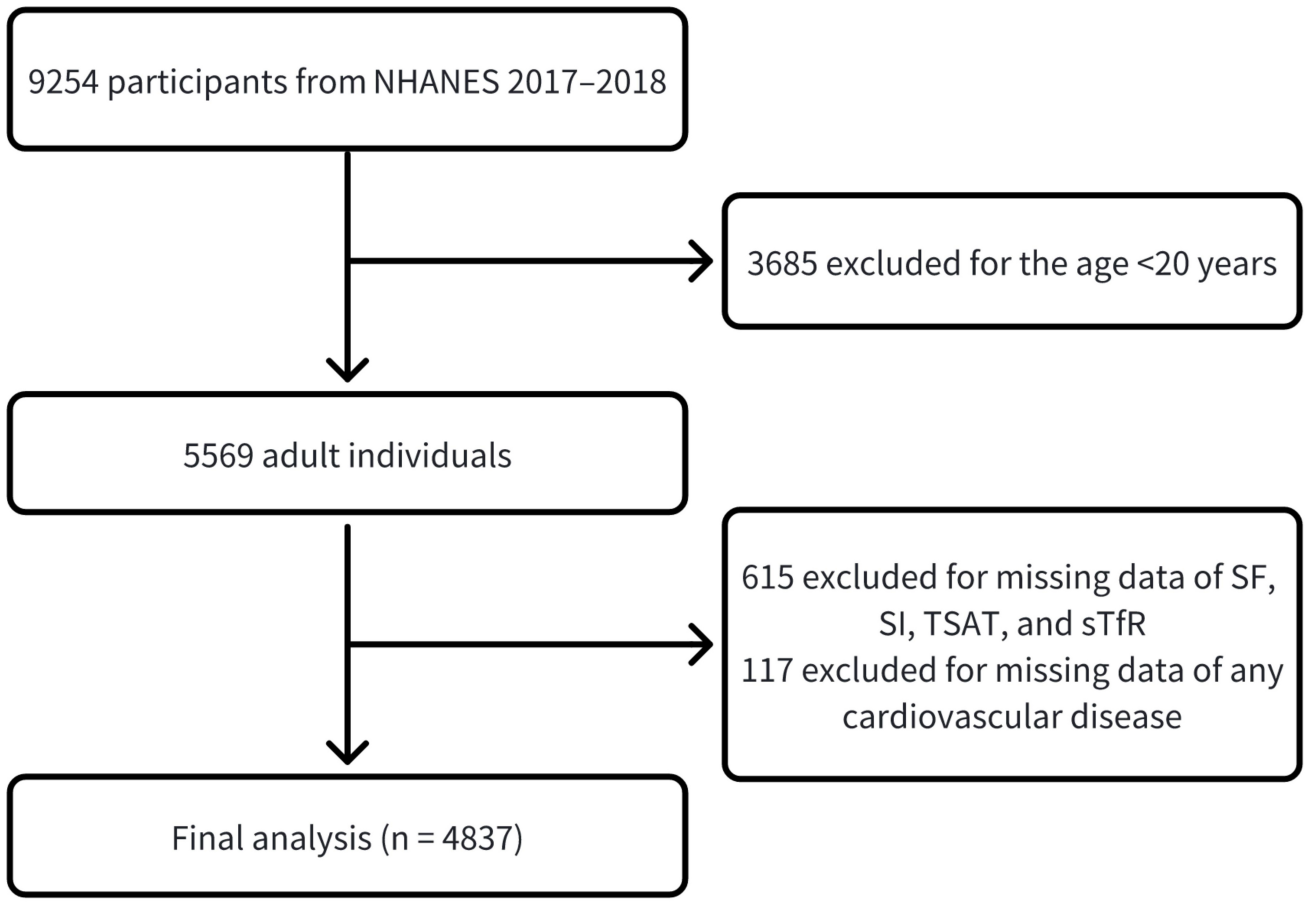
**Flow chart of study participants**. SF, serum ferritin; SI, serum iron; TSAT, transferrin saturation; sTfR, soluble transferrin receptor; NHANES, National Health and Nutrition Examination Survey.

Data were collected from the NHANES, a multistage cross-sectional study of the nonmilitary and noninstitutionalized U.S. population. Survey data were collected from the 2017–2018 NHANES. The 2017–2018 survey cycle included 9254 participants. Exclusion criteria were as follows: (a) participants aged <20 years (n = 3685); (b) missing data on SF, SI, TSAT, and sTfR (n = 615); (c) missing data on any cardiovascular disease (n = 117). In total, 4837 individuals were considered. The study flowchart is shown in Fig. 1. Information about the data is publicly available through the NHANES website.

### 2.2 Assessment for Cardiovascular Diseases

In this study, major cardiovascular events were defined as self-reported. It was a composite outcome consisting of myocardial infarction (MI), heart failure (HF), coronary heart disease (CHD), angina pectoris, or stroke [12]. Participants who responded “yes” to the following questions were regarded as CVDs: “Has a doctor or other health professional ever told you that you had a heart attack (also called myocardial infarction)/congestive heart failure/coronary heart disease/angina (also called angina pectoris)/a stroke?” (5 separate questions followed the same pattern).

### 2.3 SF, SI, TSAT, and sTfR Measurements

An immunoturbidimetric assay using a Hitachi 912 analyzer (Roche Diagnostics) was used to measure SF concentrations [13]. When the interaction between latex-bound ferritin antibodies and the antigen was examined in the sample, the antigen/antibody complexes were produced. This interaction was measured after agglutination using a turbidimetric method. Subsequently, a wavelength of 700 nm was used to measure the created complexes. Then, the complexes were normalized for the ferritin content. SI concentration was measured by a DCX-800 system (Beckman Coulter, Fullerton, CA, USA) using the timed endpoint method [14]. Using an acidic medium, ferric iron bound to transferrin was released and reduced to ferrous iron by hydroxylamine and thioglycolate. Ferrous ions quickly form a complex with ferrozine, for which changes in absorbance can be tracked at 560 nm. The resulting change in absorbance is directly proportional to the iron concentration in the sample. An immunoturbidimetric assay called particle-enhanced immunoturbidimetry was used in the study to measure sTfR [15]. An antigen–antibody reaction occurs between the antigen and anti-sTfR antibodies coated on latex particles in the sample. A photometric analysis was used to quantify the precipitate after agglutination. SI and unsaturated iron-binding capacity (UIBC) were combined to calculate the total iron-binding capacity (TIBC), and SI/TIBC × 100% was applied to calculate TSAT. The biomarkers of iron status (SI, SF, TSAT, and sTfR) were stratified into quartiles to investigate the potential relationship between iron metabolism and CVD. Detailed descriptions of measurements are available on the NHANES website.

### 2.4 Covariates

Several confounders were selected based on a previous theory and findings. The level of dietary iron intake from food was categorized by quartile distribution (<8.3 mg/d, 8.3–12.2 mg/d, 12.2–17.4 mg/d, and ≥17.4 mg/d). A daily dose of supplemented iron during the first 24 h was considered as a “yes/no” variable, and these results were divided into quartiles (0, 0.08–14 mg, 14–18 mg, 18–28 mg, or ≥28 mg). Participant demographics included age, sex, and race. Furthermore, BMI (weight/height^2^) was separated into normal/underweight (BMI <25 kg/m^2^), overweight (BMI between 25 and 30 kg/m^2^), and obese (BMI ≥30 kg/m^2^) [16]. Based on the definitions from the National Institute on Alcohol Abuse and Alcoholism (NIAAA) in the National Institute of Health, alcohol consumption was defined as none, moderate (1–2 drinks/day for men or one drink/day for women), heavy (3–4 drinks/day for men or 2–3 drinks/day for women), or binge (≥5 drinks/day for men or ≥4 drinks/day for women). Smoking status was classified as never, prior, or current. Additionally, according to the recommended Physical Activity Guidelines of ≥75 min/week of vigorous or ≥150 min/week of moderate physical activity, the participants were categorized as inactive (no physical activity), less active (< the level of recommended activity), and as physically active (≥ the level of recommended activity) [17]. The poverty-to-income ratio (PIR), which measures a family’s income level compared to the poverty threshold, was classified as <1.3, 1.3–1.8, and >1.8, and was used to measure income level. Further, the level of education was determined as “less than high school”, “high school”, or “greater than high school”.

### 2.5 Statistical Analysis

Continuous variables are expressed as the weighted mean ± SEM (standard error of the mean). Categorical variables are presented as the weighted percentage and 95% confidence interval (95% CI) and were compared using the chi-square test. Multivariable analysis was performed using the logistic regression model and reported as the odds ratio (OR). The confounding factor was selected based on clinically relevant or, when added to this model, changed the matched odds ratio on the outcome measure by at least 10% or was significantly associated with CVD. Furthermore, to ensure parsimony of the final logistic regression model, variables for inclusion were determined based on the number of events available. Model 1 was the unadjusted model. Model 2 was adjusted for demographic characteristics (age, gender, and race). Model 3 included adjustment for demographic information (age, gender, and race), individual characteristics (PIR, BMI, and level of education), health behaviors (daily alcohol consumption, physical activity status, and smoking status), comorbid conditions (history of diabetes and hypertension), laboratory tests (total cholesterol, high-density lipoprotein cholesterol, alanine aminotransferase, hypersensitive C-reactive protein, and hemoglobin), and medication and dietary history (dietary supplements of iron taken and level of dietary iron intake by food). Stratified multivariable logistic regression analysis was performed to identify target groups. Potential interactions between serum iron markers and selected risk factors for CVD (BMI, age, and gender) were performed using the likelihood-ratio test. We also performed generalized additive models (GAMs) to determine non-linear relationships between sTfR and CVD. All statistical analyses were performed using R version 4.2.3 (R Foundation for Statistical Computing, Vienna, Austria), STATA 16.0 (StataCorp, College Station, TX, USA), and Empowerstats (http://www.empowerstats.com, X&Y Solutions, Inc., Boston, MA, USA).

## 3. Results

### 3.1 Characteristics of Participants

The population-weighted characteristics of the study are summarized in Table 1. The final analysis cohort consisted of 4837 participants, of whom 48.17% were male, with an average age of 48.38 years. Compared to participants without CVDs, participants with CVDs had significantly increased levels of sTfR (*p* < 0.001) and decreased levels of SI (*p* < 0.001) and TSAT (*p* < 0.001). However, the two groups had no statistically significant difference in SF levels (*p* = 0.07). Those with CVDs tended to be predominantly male, non-Hispanic White, binge drinkers who performed no physical activity. Moreover, these participants had a higher prevalence of hypertension, obesity, and diabetes.

**Table 1.** **Weighted characteristics of participants stratified by cardiovascular diseases from the 2017–2018 NHANES**.

Characteristics		Total	Non-CVD (n = 4257)	CVD (n = 580)	*p*-value
Age (year)		48.38 ± 0.34	46.63 ± 0.36	65.20 ± 0.69	<0.001
Gender					<0.001
	Male	48.17 (46.03–50.31)	47.30 (45.03–49.58)	56.47 (50.37–62.38)	
	Female	51.83 (49.69–53.97)	52.70 (50.42–54.97)	43.53 (37.62–49.63)	
Race					<0.001
	Mexican American	8.96 (8.16–9.83)	9.49 (8.62–10.44)	3.85 (2.69–5.47)	
	Other Hispanic	7.02 (6.29–7.83)	7.33 (6.54–8.20)	4.10 (2.71–6.16)	
	Non-Hispanic White	63.06 (61.24–64.84)	62.10 (60.15–64.02)	72.20 (67.22–76.68)	
	Non-Hispanic Black	10.74 (9.98–11.56)	10.74 (9.92–11.61)	10.81 (8.72–13.33)	
	Other races	10.22 (9.24–11.29)	10.34 (9.32–11.46)	9.04 (5.97–13.47)	
Education					0.002
	Less than high school	3.76 (3.33–4.24)	3.58 (3.14–4.08)	5.44 (4.01–7.34)	
	High school or equivalent	34.60 (32.64–36.61)	33.94 (31.90–36.11)	40.59 (34.82–46.64)	
	More than high school	61.54 (59.50–63.54)	62.33 (60.18–64.44)	53.94 (47.86–59.91)	
	Not recorded	0.10 (0.05–0.23)	0.11 (0.05–0.25)	0.02 (0.00–0.18)	
Poverty–income ratio					0.006
	<1.3	17.66 (16.39–19.00)	17.44 (16.10–18.87)	19.72 (16.19–23.81)	
	1.3–1.8	8.41 (7.58–9.32)	8.02 (7.17–8.98)	12.14 (9.42–15.51)	
	≥1.8	63.49 (61.59–65.35)	64.11 (62.11–66.07)	57.56 (51.67–63.24)	
	Not recorded	10.44 (9.30–11.70)	10.42 (9.23–11.74)	10.59 (7.13–15.43)	
Daily alcohol drinking status					<0.001
	Non-drinkers	6.68 (5.75–7.76)	6.78 (5.79–7.94)	5.70 (3.61–8.90)	
	Moderate drinkers	33.80 (31.73–35.93)	34.04 (31.84–36.31)	31.46 (25.89–37.62)	
	Heavy drinkers	29.64 (27.72–31.64)	31.03 (28.97–33.16)	16.37 (12.16–21.69)	
	Binge drinkers	12.72 (11.41–14.16)	12.05 (10.69–13.56)	19.16 (14.93–24.25)	
	Not recorded	17.16 (15.74–18.67)	16.10 (14.65–17.67)	27.31 (22.31–32.95)	
Smoking status					<0.001
	Never	57.53 (55.40–59.63)	59.21 (56.95–61.42)	41.44 (35.51–47.64)	
	Prior	25.16 (23.29–27.13)	23.59 (21.63–25.66)	40.19 (34.29–46.40)	
	Current	17.31 (15.85–18.88)	17.20 (15.66–18.87)	18.36 (14.36–23.18)	
Physical activity level					0.034
	Inactive	47.15 (45.02–49.28)	46.46 (44.21–48.73)	53.69 (47.49–59.77)	
	Less active	8.54 (7.39–9.84)	8.65 (7.42–10.05)	7.51 (4.91–11.32)	
	Active	43.95 (41.83–46.10)	44.52 (42.27–46.8)	38.47 (32.56–44.75)	
	Not recorded	0.36 (0.18–0.74)	0.36 (0.17–0.80)	0.33 (0.12–0.89)	
BMI group					<0.001
	<25	25.57 (23.72–27.50)	26.51 (24.52–28.60)	16.51 (12.66–21.24)	
	25–30	30.77 (28.82–32.78)	30.77 (28.71–32.92)	30.67 (25.16–36.80)	
	≥30	42.62 (40.52–44.74)	41.92 (39.70–44.17)	49.32 (43.21–55.46)	
	Not recorded	1.05 (0.80–1.39)	0.80 (0.58–1.10)	3.49 (2.04–5.92)	
History of hypertension					<0.001
	Yes	40.06 (38.01–42.14)	36.27 (34.15–38.44)	76.36 (70.52–81.35)	
History of diabetes					<0.001
	Yes	11.59 (10.39–12.90)	8.84 (7.74–10.07)	37.95 (32.11–44.16)	
Dietary iron intake by food, mg/d					0.158
	0–8.3	20.75 (19.14–22.47)	20.66 (18.94–22.49)	21.67 (17.32–26.75)	
	8.3–12.2	23.33 (21.57–25.18)	23.62 (21.72–25.59)	20.69 (16.39–25.78)	
	12.2–17.4	24.91 (23.04–26.88)	24.56 (22.59–26.64)	28.27 (22.74–34.54)	
	≥17.4	24.33 (22.51–26.25)	24.63 (22.69–26.67)	21.54 (16.71–27.31)	
	Not recorded	6.67 (5.81–7.65)	6.55 (5.65–7.59)	7.83 (5.42–11.19)	
Daily dose of supplemented iron, mg					0.01
	None	88.12 (86.64–89.46)	88.57 (86.99–89.98)	83.82 (79.02–87.69)	
	0.08–14	2.77 (2.15–3.56)	2.76 (2.10–3.61)	2.92 (1.62–5.21)	
	14–18	0.22 (0.06–0.73)	0.24 (0.07–0.80)	0	
	18–28	5.95 (4.96–7.12)	5.67 (4.64–6.91)	8.62 (5.63–12.98)	
	≥28	2.94 (2.34–3.69)	2.76 (2.14–3.57)	4.64 (2.95–7.23)	
ALT, U/L		23.00 ± 0.38	23.20 ± 0.41	21.15 ± 0.94	0.015
ALB, g/L		4.08 ± 0.01	4.09 ± 0.01	3.95 ± 0.02	<0.001
BUN, mg/dL		14.93 ± 0.10	14.59 ± 0.11	18.20 ± 0.40	<0.001
Scr, mg/dL		0.88 ± 0.01	0.87 ± 0.01	1.03 ± 0.02	<0.001
HGB, g/dL		14.19 ± 0.03	14.22 ± 0.03	13.90 ± 0.10	<0.001
HbA1c, %		5.69 ± 0.02	5.63 ± 0.02	6.20 ± 0.06	<0.001
hs-CRP, mg/L		3.88 ± 0.14	3.67 ± 0.13	5.90 ± 0.70	<0.001
SF, µg/L		148.42 ± 3.04	147.02 ± 3.23	161.84 ± 8.95	0.07
SI, µmol/L		15.75 ± 0.15	15.90 ± 0.16	14.29 ± 0.36	<0.001
TSAT, %		27.48 ± 0.24	27.66 ± 0.25	25.78 ± 0.66	<0.001
sTfR, mg/L		3.20 ± 0.03	3.14 ± 0.03	3.79 ± 0.12	<0.001
TC, mmol/L		4.89 ± 0.02	4.93 ± 0.02	4.51 ± 0.08	<0.001
HDL-C, mmol/L		1.39 ± 0.01	1.40 ± 0.01	1.30 ± 0.03	<0.001
TG, mmol/L					<0.001
	0.113–0.7	12.32 (10.93–13.87)	13.01 (11.50–14.69)	5.70 (3.49–9.18)	
	0.7–1.061	12.69 (11.31–14.22)	12.82 (11.35–14.45)	11.46 (8.15–15.88)	
	1.061–1.535	10.86 (9.65–12.19)	10.59 (9.31–12.01)	13.45 (10.12–17.65)	
	≥1.535	12.16 (10.85–13.62)	11.78 (10.40–13.30)	15.89 (11.79–21.07)	
	Not recorded	51.96 (49.82–54.09)	51.84 (49.52–54.07)	53.54 (47.37–59.53)	
LDL-C, mmol/L					<0.001
	0.465–2.224	11.30 (10.00–12.74)	10.32 (9.00–11.82)	20.64 (15.97–26.24)	
	2.224–2.767	12.15 (10.82–13.61)	12.71 (11.27–14.30)	6.78 (4.83–9.43)	
	2.767–3.414	12.15 (10.78–13.66)	12.69 (11.20–14.33)	6.99 (4.73–10.21)	
	≥3.414	11.94 (10.64–13.37)	11.98 (10.61–13.50)	11.49 (8.05–16.15)	
	Not recorded	52.47 (50.33–54.60)	52.35 (50.02–54.57)	54.11 (47.97–60.12)	

Values are the weighted mean ± SEM or weighted % (95% confidence interval). *p*-values are weighted. CVD, cardiovascular disease; BMI, body mass index; ALT, alanine aminotransferase; ALB, albumin; BUN, blood-urea-nitrogen; Scr, serum creatinine; HGB, hemoglobin; HbA1c, glycated hemoglobin A1c; hs-CRP, hypersensitive C-reactive protein; SF, serum ferritin; SI, serum iron; TSAT, transferrin saturation; sTfR, soluble transferrin receptor; TG, triglycerides; TC, total cholesterol; HDL-C, high-density lipoprotein-cholesterol; LDL-C, low-density lipoprotein-cholesterol; NHANES, National Health and Nutrition Examination Survey.

### 3.2 Association between Iron Status and CVDs

Associations of iron metabolism index levels with the risk of CVDs following analysis by multivariable logistic regression models are presented in Table 2. For SI, compared with the lowest quartile group, participants from the highest quartile group experienced a 28% lower risk for CVDs in the non-adjusted model (OR 0.72, 95% CI 0.55–0.93, *p* < 0.05). However, SF and sTfR showed increased risks of CVDs (Q1 as a reference, Q4 OR 1.6, 95% CI 1.25–2.07, *p* < 0.001; Q4 OR 2.75, 95% CI 2.11–3.58, *p* < 0.001, respectively). After adjusting for potential confounding factors (Model 3), compared with the lowest quartile group, the ORs with 95% CIs for CVD risks were 1.18 (0.89, 1.56), 1.04 (0.77, 1.4), and 0.87 (0.62, 1.2) for SI; 1.02 (0.75, 1.39), 1.01 (0.74, 1.38), and 1.07 (0.78, 1.47) for SF; 1.32 (0.98, 1.79), 1.18 (0.86, 1.61), and 1.05 (0.75, 1.47) for TSAT; 1.35 (0.98, 1.87), 1.8 (1.32, 2.46), and 2.1 (1.54, 2.87) for sTfR in the second, third, and highest quartile groups, respectively. Only sTfR levels had a significant positive association with CVD risks.

**Table 2.** **Associations between iron metabolism and CVD from the 2017–2018 NHANES**.

		Model 1	Model 2	Model 3
		OR (95% CI), *p* for trend	OR (95% CI), *p* for trend	OR (95% CI), *p* for trend
SI (µmol/L)		0.002	<0.001	0.259
	Q1 (<11.1)	Reference	Reference	Reference
	Q2 (11.1–14.7)	1.20 (0.95, 1.52)	0.97 (0.75, 1.25)	1.18 (0.89, 1.56)
	Q3 (14.7–18.8)	0.93 (0.73, 1.19)	0.73 (0.56, 0.95) *	1.04 (0.77, 1.40)
	Q4 (≥18.8)	0.72 (0.55, 0.93) *	0.61 (0.46, 0.81) **	0.87 (0.62, 1.20)
SF (µg/L)		<0.001	0.427	0.696
	Q1 (<51.3)	Reference	Reference	Reference
	Q2 (51.3–109)	1.33 (1.02, 1.72) *	0.87 (0.65, 1.15)	1.02 (0.75, 1.39)
	Q3 (109–199)	1.42 (1.09, 1.83) **	0.83 (0.63, 1.10)	1.01 (0.74, 1.38)
	Q4 (≥199)	1.60 (1.25, 2.07) ***	0.88 (0.66, 1.17)	1.07 (0.78, 1.47)
TSAT (%)		0.368	0.001	0.818
	Q1 (<19)	Reference	Reference	Reference
	Q2 (19–26)	1.36 (1.06, 1.75) *	0.98 (0.75, 1.29)	1.32 (0.98, 1.79)
	Q3 (26–34)	1.17 (0.90, 1.51)	0.79 (0.60, 1.04)	1.18 (0.86, 1.61)
	Q4 (≥34)	0.96 (0.73, 1.25)	0.66 (0.49, 0.89) **	1.05 (0.75, 1.47)
sTfR (mg/L)		<0.001	<0.001	<0.001
	Q1 (<2.47)	Reference	Reference	Reference
	Q2 (2.47–2.97)	1.40 (1.05, 1.87) *	1.25 (0.92, 1.70)	1.35 (0.98, 1.87)
	Q3 (2.97–3.68)	2.11 (1.60, 2.77) ***	1.78 (1.32, 2.38) ***	1.80 (1.32, 2.46) ***
	Q4 (≥3.68)	2.75 (2.11, 3.58) ***	2.41 (1.80, 3.22) ***	2.10 (1.54, 2.87) ***

Abbreviations are the same as those in Table 1. Model 1: non-adjusted model; Model 2: adjusted for age, gender, race; Model 3: adjusted for age, gender, race, education, poverty income ratio, BMI, alcohol drinking status, smoking status, physical activity level, diabetes, hypertension, dietary iron intake by food, daily iron supplement, total cholesterol, high-density lipoprotein-cholesterol, ALT, hs-CRP, and hemoglobin. *p < 0.05, **p < 0.01, ***p < 0.001. *p*-values were calculated using Q1 as the reference. *p* for the trend is presented as the differences between Q1, Q2, Q3, and Q4.

### 3.3 Modification of BMI on Iron Status and CVDs

Furthermore, we performed an interaction analysis between the iron metabolism index levels and CVD stratified by BMI (<25 kg/m^2^; ≥25 to <30 kg/m^2^; ≥30 kg/m^2^). Significant interaction terms with BMI were only observed between sTfR and CVD (*p*-interaction = 0.042, Table 3). The association between sTfR and CVD risk differed in the BMI subgroup. Following multivariable logistic regression, the sTfR levels were significantly positively correlated with CVD risks in overweight and normal/underweight adults rather than obese adults. Quartile analyses in the overweight group discovered that using a reference of the first quartile group of sTfR, participants in the second, third, and highest quartile groups had 83% (OR, 1.83 [1.02, 3.28]), 143% (OR, 2.43 [1.37, 4.33]), and 264% (OR, 3.64 [2.07, 6.39]) higher odds of CVD risk. The OR values showed a tendency for higher risks of CVDs in the higher sTfR quartiles compared with the lowest quartile group. In the normal/underweight group, participants in the highest quartile sTfR group also had 241% (OR, 3.41 [1.6, 7.28]) higher CVD risk odds than those in the first quartile group. However, no relationship was observed between the sTfR level and CVD risks in the obese group in the quartile analyses. Moreover, we conducted subgroup analyses based on gender. The interaction effect for gender between iron biomarkers and CVD was non-significant. Subgroup analysis was also performed according to age categories (<60 years and ≥60 years). Age yielded a significant interaction between SF and CVD.

**Table 3.** **Subgroup analysis between iron metabolism and CVD stratified by BMI, gender, and age among US adults from the 2017–2018 NHANES**.

		BMI <25	BMI ≥25, <30	BMI ≥30	*p*-interaction	Male	Female	*p*-interaction	<60 yrs	≥60 yrs	*p*-interaction
		OR (95% CI)	OR (95% CI)	OR (95% CI)		OR (95% CI)	OR (95% CI)		OR (95% CI)	OR (95% CI)	
SI (µmol/L)					0.416			0.337			0.284
	Q1 (<11.1)	Reference	Reference	Reference		Reference	Reference		Reference	Reference	
	Q2 (11.1–14.7)	1.33 (0.65, 2.71)	1.06 (0.61, 1.84)	1.23 (0.82, 1.84)		1.20 (0.79, 1.82)	1.19 (0.80, 1.76)		0.79 (0.46, 1.34)	1.38 (0.99, 1.93)	
	Q3 (14.7–18.8)	1.93 (0.92, 4.08)	0.79 (0.44, 1.40)	0.96 (0.62, 1.50)		1.30 (0.85, 1.98)	0.80 (0.51, 1.27)		0.98 (0.58, 1.67)	1.06 (0.74, 1.52)	
	Q4 (≥18.8)	1.06 (0.49, 2.29)	0.67 (0.37, 1.21)	0.98 (0.59, 1.62)		0.90 (0.58, 1.41)	0.82 (0.49, 1.36)		0.72 (0.40, 1.29)	0.97 (0.65, 1.44)	
SF (µg/L)					0.082			0.303			0.046
	Q1 (<51.3)	Reference	Reference	Reference		Reference	Reference		Reference	Reference	
	Q2 (51.3–109)	1.08 (0.54, 2.18)	0.78 (0.45, 1.36)	1.16 (0.71, 1.89)		1.30 (0.80, 2.11)	0.81 (0.53, 1.24)		1.57 (0.86, 2.87)	0.94 (0.66, 1.36)	
	Q3 (109–199)	0.58 (0.27, 1.25)	0.83 (0.47, 1.46)	1.41 (0.88, 2.27)		1.17 (0.72, 1.89)	0.90 (0.58, 1.40)		1.68 (0.90, 3.16)	0.98 (0.69, 1.41)	
	Q4 (≥199)	0.65 (0.30, 1.42)	1.01 (0.58, 1.73)	1.35 (0.82, 2.23)		1.10 (0.68, 1.76)	1.13 (0.70, 1.83)		2.65 (1.39, 5.09) **	0.94 (0.65, 1.35)	
TSAT (%)					0.456			0.753			0.463
	Q1 (<19)	Reference	Reference	Reference		Reference	Reference		Reference	Reference	
	Q2 (19–26)	0.87 (0.40, 1.88)	1.17 (0.66, 2.07)	1.67 (1.08, 2.59) *		1.21 (0.76, 1.92)	1.42 (0.94, 2.15)		1.05 (0.60, 1.84)	1.45 (1.01, 2.08)	
	Q3 (26–34)	1.07 (0.50, 2.29)	0.87 (0.48, 1.55)	1.44 (0.90, 2.30)		1.06 (0.67, 1.69)	1.34 (0.86, 2.09)		1.29 (0.74, 2.24)	1.13 (0.77, 1.65)	
	Q4 (≥34)	0.86 (0.39, 1.91)	0.74 (0.40, 1.36)	1.37 (0.83, 2.30)		1.02 (0.63, 1.64)	0.96 (0.57, 1.60)		1.19 (0.64, 2.19)	1.12 (0.75, 1.68)	
sTfR (mg/L)					0.042			0.815			0.108
	Q1 (<2.47)	Reference	Reference	Reference		Reference	Reference		Reference	Reference	
	Q2 (2.47–2.97)	2.25 (1.09, 4.63) *	1.83 (1.02, 3.28) *	0.86 (0.51, 1.44)		1.28 (0.84, 1.96)	1.34 (0.80, 2.26)		1.60 (0.92, 2.78)	1.41 (0.95, 2.09)	
	Q3 (2.97–3.68)	2.40 (1.17, 4.93) *	2.43 (1.37, 4.33) **	1.24 (0.77, 1.97)		1.95 (1.30, 2.92) **	1.60 (0.96, 2.64)		1.20 (0.67, 2.14)	2.24 (1.54, 3.25) ***	
	Q4 (≥3.68)	3.41 (1.60, 7.28) **	3.64 (2.07, 6.39) ***	1.28 (0.80, 2.04)		2.25 (1.51, 3.36) ***	1.81 (1.09, 3.02) *		1.55 (0.88, 2.75)	2.46 (1.69, 3.57) ***	

Abbreviations are the same as those in Table 1. Adjusted for age, gender, race, education, poverty income ratio, BMI, alcohol drinking status, smoking status, physical activity level, diabetes, hypertension, dietary iron intake from food, daily iron supplement, total cholesterol, high-density lipoprotein-cholesterol, ALT, hs-CRP, and hemoglobin. *p < 0.05, **p < 0.01, ***p < 0.001. *p*-values were calculated using Q1 as the reference. The strata variable was not included when stratifying by itself.

### 3.4 Detection of Non-linear Relationships

**Fig. 2.**
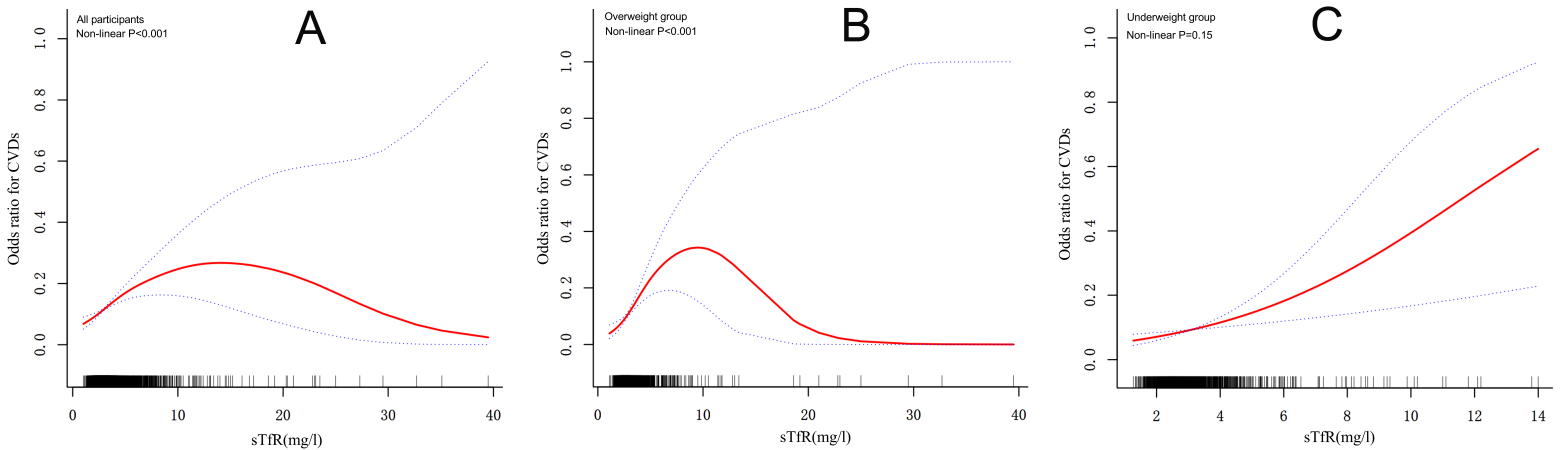
**Association between sTfR and the risk of CVD in all participants (A), the group of overweight individuals (B), and the group of underweight individuals (C)**. The red line and dashed line represent the estimated values and their corresponding 95% confidence interval. Analyses were adjusted for age, race, gender, PIR, BMI (only for all participants), level of education, daily alcohol consumption, physical activity status, smoking status, history of diabetes and hypertension, total cholesterol, high-density lipoprotein-cholesterol, alanine aminotransferase, hypersensitive C-reactive protein, hemoglobin, dietary supplements of iron taken, and level of dietary iron intake from food. sTfR, soluble transferrin receptor; CVD, cardiovascular disease; PIR, poverty-to-income ratio; BMI, body mass index.

To further clarify the relationships between the sTfR and the risks of CVDs, we employed an adjusted smooth curve plot model and stratified subgroup analysis by BMI. There was an inverted U-shaped relationship between the sTfR and the CVD risks in the total and overweight groups (*p* < 0.001 for log-likelihood ratio; Fig. 2A,B). We discovered that the inflection points for total participants were 4.44 mg/L and 5.35 mg/L for overweight participants (Table 4). When the sTfR level was less than 4.44 mg/L or 5.35 mg/L, the per unit increase in the sTfR level was correlated with a 42% and a 78% greater adjusted OR of CVD risk, respectively (total group: OR, 1.42 [1.24, 1.63]; overweight group: OR, 1.78 [1.44, 2.19]). No relationship was observed when the sTfR level exceeded the turning point. However, the sTfR level was linearly associated with the CVD risk in the normal/underweight group (*p* = 0.15 for log-likelihood ratio; Fig. 2C). A per unit increase in the sTfR level was correlated with a 31% greater adjusted OR of CVD risk (OR, 1.31 [1.1, 1.55]).

**Table 4.** **Threshold effect analysis of sTfR on risks of CVD in the total population and stratified by BMI from the 2017–2018 NHANES**.

	Adjusted OR (95% CI), *p*-value		
CVD	All participants	Overweight group	Underweight group
Fitting by the standard linear model	1.09 (1.04, 1.15) <0.001	1.07 (0.99, 1.15) 0.065	1.31 (1.10, 1.55) 0.002
Fitting by two-piecewise Cox proportional risk model			
Inflection point	4.44 mg/L	5.35 mg/L	2.6 mg/L
sTfR < inflection point	1.42 (1.24, 1.63) <0.001	1.78 (1.44, 2.19) <0.001	3.18 (0.89, 11.42) 0.076
sTfR ≥ inflection point	1.01 (0.94, 1.08) 0.868	0.88 (0.73, 1.05) 0.161	1.23 (1.01, 1.50) 0.041
*p* for log-likelihood ratio	<0.001	<0.001	0.150

Abbreviations are the same as those in Table 1. Adjusted for age, gender, race, education, poverty income ratio, BMI (only for all participants), alcohol drinking status, smoking status, physical activity level, diabetes, hypertension, dietary iron intake from food, daily iron supplement, total cholesterol, high-density lipoprotein-cholesterol, ALT, hs-CRP, and hemoglobin.

## 4. Discussion

This cross-sectional study examined the relationship between iron metabolism levels and CVD risks in a large-scale U.S. general population. Based on the NHANES database, the increased sTfR levels were positively associated with the CVD risk, and the BMI varied the association. Adjusting for potential confounders did not alter this association. The most important finding of our study was that sTfR had an inverted U-shaped association with CVD risk in the overweight group. In light of these results, adding BMI could significantly improve the predictive value of iron status for predicting the risk of CVD. Nevertheless, this study did not show that the SF, SI, or TSAT levels were correlated with the risk of CVDs after adjusting for relevant confounders. To our knowledge, this is the first nationally representative study to investigate the potential modification of BMI on the relationship between sTfR and CVDs.

### 4.1 Markers of Iron Status

Iron plays an essential role in supporting physical functions in the human body. However, the bioavailable forms of iron are subject to its ability to produce highly insoluble oxides. ID is a widespread nutritional disorder affecting billions of people worldwide [18]. ID and iron overload are some of the most common causes of human diseases due to their disruption of iron homeostasis [19]. Iron is delivered into cells by binding with transferrin and internalization through its receptor. Circulating SF and TSAT concentrations reflect tissue and serum iron load. SF is a major intracellular iron storage protein [20]. Serum concentrations <30 mg/L are commonly used to determine ID [21]. However, SF also frequently increases in response to inflammation [21, 22], which implies that a standard SF could be misleading. TSAT is also an acute-phase reactant that can be decreased during inflammation [23]. In contrast to SF, sTfR concentrations are not influenced by inflammation or infection [24].

### 4.2 Impact of sTfR on CVD Risk

High sTfR concentrations, reflecting decreased metabolic-related iron, were found to be related to poor clinical outcomes among patients with HF [25] or diabetic patients with CHD [10]. The powerful predictive ability of high sTfR, in contrast with the relatively weak predictive capability of low SF, low TSAT, and low SI, suggests that deficiency in cellular functions and iron-limited erythropoiesis are significant cornerstones [26]. SI and TSAT were found to be inversely associated with the prevalence of CVD [7, 27]. As a result of their broad diurnal variation and low intraclass correlation, these biomarkers may not be accurate measures of body iron stores [28, 29]. A reverse causal relationship could also exist between iron stores and inflammation, with inflammation affecting body iron stores [27]. Inflammation always occurs with the occurrence and development of CVD [30]. Inflammation has been proven to reduce SI and TSAT [31, 32]. There has been evidence that sTfR is a sensitive marker for ID since it is a truncated form of the tissue transferrin receptor [33]. Thus, ID may result in an overexpression of sTfR concentrations [34]. Interestingly, the repletion of iron also restored the performance of sTfR levels to normal levels [10, 35]. Recently, some studies suggested that elevated sTfR was associated with cardiovascular diseases, such as CHD and HF [15, 36, 37]. Indeed, Zhu S *et al*. [15] found that sTfR had a significant positive relationship with CVDs, data that is consistent with our result.

### 4.3 Modification of BMI on sTfR and CVDs

Nevertheless, unlike the present study, no previous studies have focused on BMI modifications on iron status and CVDs. Weight gain has long been regarded as a risk factor for CVDs. Moreover, since it represents a possible cause of metabolic diseases, obesity has been viewed as an imbalance between energy intake and output [38]. Obesity-related diabetes, non-alcoholic fatty liver disease, and hypertension are recognized CVD risk factors [39]. We performed a stratified analysis using BMI to investigate the effects of sTfR on CVDs, and the findings demonstrated that these results might differ across groups and might have exacerbated the apparent differences between groups. We explored an inverted U-shaped association between the sTfR and the CVD risks in the total population. Furthermore, subgroup analyses revealed that this result was primarily attributed to findings for overweight participants. It was non-linear and significantly different from the linear association observed in the normal/underweight group. From this curve, the incidence of CVDs was elevated with an increase in sTfR up to a certain point, beyond which the incidence did not rise further. One mechanism may explain why the higher risks of CVD were found on the left side of the inflection point in the overweight participants. ID resulted in an overexpression of sTfR concentrations. In this case, elevated sTfR concentrations, reflecting decreased metabolic-related iron, were found to be associated with the high prevalence of CVD. After a certain point, the impact of ID-related sTfR elevation tended to be saturated. It is also likely that other factors may contributed to the abnormal sTfR elevation. Overweight individuals in the population face a variety of health risks; it has been shown that sTfR is overexpressed in most tumors [40, 41, 42]. However, it is possible that the elevated sTfR may not be a true representation of ID in this case. These factors seem to attenuate the influence of sTfR elevation on CVD risks. Our results implied that sTfR levels were significantly related to higher CVD risks within a specific range. These findings could promote dietary preventive strategies through cooperating with clinicians to decrease morbidity related to CVDs. Identifying a target population at higher risk of CVDs in patients with ID might have substantial public health implications.

### 4.4 Potential Mechanisms of sTfR on CVDs

Iron is an essential trace metal required for cellular growth and metabolism. The primary mechanism of iron biotoxicity is the participation of excess ferrous iron (Fe^2+^), which is involved in the Fenton reaction [43]. Fe^2+^ associated with hydrogen peroxide or oxygen catalyzes large amounts of ROS production, contributing to lipid peroxidation and organ damage [44]. Thus, iron has been implicated in the pathological mechanisms of hemochromatosis, cancer, and CVD [45]. In addition, hypoxia in myocardial cells plays a significant role in the development of CVD [46]. It has been demonstrated that hypoxia-induced factors stimulate the expression of sTfR in hypoxic cells [47, 48]. sTfR may serve as an indicator of cellular oxygen requirements [49]. Thus, hypoxia may be the reason why sTfR in CVD patients is significantly increased. Furthermore, individuals are more likely to suffer from CVD due to increased blood glucose levels, insulin resistance, inflammation, and reactive oxygen species [50]. Several lines of evidence suggest that inflammatory cytokines and reactive oxygen species can regulate tissue transferrin receptor expression [51, 52]. In our study, the CRP levels were significantly higher in patients with CVD compared with healthy controls, which was in line with a previous study [53]. Elevated CRP levels indicated systemic inflammatory responses. The inflammatory state induces the release of hepcidin, the iron-regulating hormone, thereby altering iron transport and cellular iron metabolism [54, 55]. Hepcidin, as a marker of incident CVD in primary-prevention settings, has been strongly supported in various studies. Hepcidin levels were strongly raised during myocarditis and myocardial ischemia [56, 57]. There is an inter-relationship between inflammation, hepcidin, and iron metabolism [58]. Studies conducted *in vitro* and *in vivo* suggest that insulin may also regulate the expression of tissue transferrin receptors [59, 60], whose levels are linked to inflammation in insulin-resistant individuals [61]. Despite the need for more evidence, sTfR concentrations may also be determined by other factors. Whether sTfR reflects body iron status in a wider setting remains unknown, especially when insulin resistance exists.

### 4.5 Limitations

The present cross-sectional study recruited a significant proportion of adult participants, comprehensively investigating the association between biomarkers of iron metabolism and CVDs. Notably, our results demonstrated an inverted U-shaped association between sTfR and CVD risks in the overweight participants, which may provide primary management for suitable iron supplementation of patients with CVDs. However, several limitations should be addressed. First, the NHANES study design is cross-sectional, limiting the assessment of causal relationships. Second, the NHANES uses self-reported data rather than official hospital records, which may have been influenced by recall bias. Third, residual confounding from unmeasured covariates or measurement errors cannot be excluded even after adjusting for several critical potential covariates in the analyses. Future prospective studies with more comprehensive variables and multiple longitudinal assessments must elucidate the underlying mechanism between iron metabolism biomarkers, BMI, and CVD.

## 5. Conclusions

In conclusion, this nationwide, population-based study demonstrated that the increased sTfR levels may be positively related to the CVD risk, and BMI may modify the association. We further discovered an inverted U-shaped association between sTfR levels and CVD risks in the overweight participants. The threshold of 5.35 mg/L is a potential target for prevention measures to lower the risk for CVDs. These observations highlight the potential significance of evaluating and monitoring sTfR status in preventing CVD among overweight adults.

## Data Availability

Data are available upon reasonable request (contact the corresponding author, Dr. Yang Gu).

## Abbreviations

CVD, cardiovascular disease; BMI, body mass index; ALT, alanine aminotransferase; ALB, albumin; BUN, blood-urea-nitrogen; Scr, serum creatinine; HGB, hemoglobin; HbA1c, glycated hemoglobin A1c; hs-CRP, hypersensitive C-reactive protein; SF, serum ferritin; SI, serum iron; TSAT, transferrin saturation; sTfR, soluble transferrin receptor; TG, triglycerides; TC, total cholesterol; HDL-C, high-density lipoprotein-cholesterol; LDL-C, low-density lipoprotein-cholesterol; NHANES, National Health and Nutrition Examination Survey.
